# MicroRNAs and Drug Addiction

**DOI:** 10.3389/fgene.2013.00043

**Published:** 2013-05-10

**Authors:** Purva Bali, Paul J. Kenny

**Affiliations:** ^1^Laboratory of Behavioral and Molecular Neuroscience, Department of Molecular Therapeutics, The Scripps Research Institute – FloridaJupiter, FL, USA; ^2^Laboratory of Behavioral and Molecular Neuroscience, Department of Neuroscience, The Scripps Research Institute – FloridaJupiter, FL, USA

**Keywords:** miRNA, miR-212, MeCP2, cocaine

## Abstract

Drug addiction is considered a disorder of neuroplasticity in brain reward and cognition systems resulting from aberrant activation of gene expression programs in response to prolonged drug consumption. Non-coding RNAs (ncRNAs) are key regulators of almost all aspects of cellular physiology. MicroRNAs (miRNAs) are small (∼21–23 nucleotides) ncRNAs transcripts that regulate gene expression at the post-transcriptional level. Recently, miRNAs were shown to play key roles in the drug-induced remodeling of brain reward systems that likely drives the emergence of addiction. Here, we review evidence suggesting that one particular miRNA, miR-212, plays a particularly prominent role in vulnerability to cocaine addiction. We review evidence showing that miR-212 expression is increased in the dorsal striatum of rats that show compulsive-like cocaine-taking behaviors. Increases in miR-212 expression appear to protect against cocaine addiction, as virus-mediated striatal miR-212 overexpression decreases cocaine consumption in rats. Conversely, disruption of striatal miR-212 signaling using an antisense oligonucleotide increases cocaine intake. We also review data that identify two mechanisms by which miR-212 may regulate cocaine intake. First, miR-212 has been shown to amplify striatal cAMP response element binding protein (CREB) signaling through a mechanism involving activation of Raf1 kinase. Second, miR-212 was also shown to regulate cocaine intake by repressing striatal expression of methyl CpG binding protein 2 (MeCP2), consequently decreasing protein levels of brain-derived neurotrophic factor (BDNF). The concerted actions of miR-212 on striatal CREB and MeCP2/BDNF activity greatly attenuate the motivational effects of cocaine. These findings highlight the unique role for miRNAs in simultaneously controlling multiple signaling cascades implicated in addiction.

## Introduction

Non-coding RNAs (ncRNAs) can be defined as biologically functional RNAs that do not encode proteins. This class of transcripts is characterized by the presence of an increased density of stop codons and lack of extensive open reading frames (ORFs). For decades, work on ncRNAs focused almost exclusively on transport RNAs (tRNAs) and ribosomal RNAs (rRNAs), which are key regulators of mRNA translation into encoded proteins. However, recent advances in high throughput sequencing technologies have revealed tremendous diversity in ncRNAs. Moreover, ncRNAs are playing an increasingly more recognized role in key aspects of cellular function, including the regulation of gene expression, often through novel mechanisms of action. ncRNAs have also been implicated in key cellular process such as DNA imprinting, RNA splicing, editing, transcription, mRNA degradation, and translational repression. Interestingly, analysis of ncRNA complexity through evolution reveals that the proportion of non-coding sequences in eukaryotic genomes correlates closely with the complexity of the organism, even when proportion of the protein coding genes remains relatively static across organisms (Heimberg et al., 2008).

It is currently thought that only about 2% of the human genome codes for functional proteins, yet more than 80% of transcripts encoded in the genome may have some biochemical activity. In a recent study, Djebali et al. (2012) used ultra-deep sequencing of RNAs from different cell lines and concluded that ∼75% of the genome is transcribed at some point during the cellular life cycle. These extensive studies using sequencing and sophisticated ncRNA prediction algorithms have led to the identification of thousands of ncRNAs in the human genome, and perhaps many more remain to be discovered (Majer and Booth, 2010; Yang et al., 2010).

On the basis of their length and function this heterogeneous group of transcripts can be further categorized into short ncRNAs and long ncRNAs. The short ncRNAs include microRNAs (miRNAs) and short-interfering RNAs (siRNAs), which are generally 18–25 nucleotides in length. Small RNAs (smRNAs) such as the small nucleolar RNAs (snoRNAs), smRNAs, piwi-interacting RNA (piRNAs) are between 20 and 300 nucleotides in length. The long ncRNAs (>200 nucleotides) can further be grouped into different categories on the basis of their origin (Orom and Shiekhattar, 2011) and have a broader spectrum of functions including chromatin modulation, recruitment of transcription factors, and nuclear-cytoplasmic transport (Mattick and Makunin, 2006). Indeed, there is now compelling computational and experimental evidence that many previously uncharacterized ncRNAs of unknown function, considered largely “transcriptional noise,” have key functional roles in almost all aspects of cellular physiology.

Using the high throughput *in situ* expression data from Allen Brain Atlas, Mercer et al. have recently shown that more than 800 different ncRNAs are widely expressed in the adult brain (Mercer et al., 2008). In another study, Landgraf et al. created a miRNA expression atlas by sequencing and analyzing multiple smRNAs libraries (Landgraf et al., 2007). Their data also reveals a tissue/cell specific expression of these RNAs, which correlates with *in situ* expression data. Hence, it is likely that ncRNAs play an important role in basic aspects of brain function and behavior.

## MicroRNAs in Brain

Amongst the different classes of ncRNAs, miRNAs are perhaps the best characterized. Since their discovery in *C. elegans* in 1993 (Lee et al., 1993), hundreds of miRNAs have been identified in different species. Initial studies demonstrated that these short (∼22 nucleotide) RNAs are mainly involved in binding to the 3′ untranslated region (3′UTR) of mRNA transcripts that share complementarity with nucleotides in their so-called seed sequence (nucleotides 2–7). Binding of miRNAs to mRNA transcripts results in post-transcription silencing of the target mRNA *via* RNA-induced silencing complex (RISC)-induced translational repression or sequestering them for storage or degradation (Bartel, 2004). However, emerging new evidence suggests that the non-seed region of miRNAs may bind to the 5′UTR or the coding sequence of target transcripts and thereby influence translation processes (Orom et al., 2008; Elcheva et al., 2009). To add further complexity, recent reports show that miRNAs can also positively regulate gene expression in some cellular contexts (Vasudevan et al., 2007; Place et al., 2008). Computational sequence analysis predicts that each miRNA can target 10–100s of mRNA transcripts (Esteller, 2011). In addition, each gene transcript can itself be targeted by potentially hundreds of miRNAs.

The brain may be a particularly prominent organ within which miRNAs play an important role in controlling gene expression and neuronal activity. In a study by Miska et al. it was established that there was considerable abundance of miRNAs in rodent and primate brain (Miska et al., 2004). Further, they developed a microarray-based method to monitor the spatiotemporal expression of miRNAs during mouse brain development and found that there was a dynamic change in miRNA expression levels during development. As a result of these new methodologies there have been more than 300 miRNAs identified in adult mouse brain. Of these brain-enriched miRNAs some are present ubiquitously whereas others are expressed in a cell specific manner (Bak et al., 2008).

Another powerful tool that improved our understanding of the functional relevance of miRNAs in neuronal tissues was the development of mice with genetic manipulations in the genes encoding Dicer and Arognaute2 (Ago2). Both of these proteins are important components of the miRNA biogenesis pathway. Dicer is an endoribonuclease that catalyzes the processing of precursor miRNA into its mature form. Characterization of Dicer1 mutants in *C. elegans* suggested that this protein plays a critical role in brain development (Grishok et al., 2001). To assess its role in mammalian brain development Bernstein et al. disrupted Dicer in mice and observed embryonic lethality with depleted number of stem cells, suggesting that this enzyme is important for development in general (Bernstein et al., 2003). Subsequently a number of groups knocked down Dicer *via* conditional gene targeting, with the findings consistent in demonstrating that ablation of dicer results in decreased miRNA expression and defects in neuronal cell differentiation and survival (Kanellopoulou et al., 2005; Murchison et al., 2005; Schaefer et al., 2007). These phenotypes could be rescued by re-expression of the gene (Kanellopoulou et al., 2005), highlighting the importance for Dicer and the smRNAs species processed by this endoribonuclease in neuronal development and function. More recently, conditional deletion of Dicer established that miRNAs expressed during neurogenesis play a role in the maintenance of neural progenitor cells, with disruption in this action contributing to defects in neuronal migration and subsequently in cortical lamination (De Pietri Tonelli et al., 2008; Kawase-Koga et al., 2009; Clovis et al., 2012).

Arognaute2, a member of the argonaute family of proteins, is the catalytically active component of the RISC that not only facilitates miRNA processing but also their regulatory actions on target mRNA transcripts (Hock and Meister, 2008). In a study by Diederichs and Haber, it was shown that overexpression of Ago2 results in increased miRNA biogenesis (Diederichs and Haber, 2007). Similar to genetic disruption of Dicer, Ago2 deletion results in embryonic lethality in mice, but conditional knock down of Ago2 reveals defects in neural tube closure and mispatterning of the anterior structure of the brain. Mouse embryonic fibroblast (MEFs) isolated from these mutant mice have reduced miRNA expression, which can be reversed by Ago2 expression (Morita et al., 2007). These findings highlight the importance of Ago2 in neuronal differentiation, brain morphogenesis, and development (Liu et al., 2004; Morita et al., 2007).

Schratt et al. found that the brain-specific miRNA, miR-134, is localized to the synaptodendritic compartment and negatively regulates the size of dendritic spines (Schratt et al., 2006). This action of miR-134 occurs through its inhibitory action on lim1 kinase expression. Another brain-enriched miRNA, miR-124, has been shown to play a critical role in the transition of progenitor neuronal cells to adult neurons by inhibiting networks of non-neuronal genes, thereby facilitating the expression of the neuronal identity (Conaco et al., 2006; Makeyev et al., 2007). Aizawa and colleagues examined the effects of double deletion of miR-9-2 and miR-9-3 on brain development in mice (Shibata et al., 2008, 2011). They found that these miR-9 family members regulate the proliferation and differentiation of neural progenitor cells in telencephalon through inhibitory actions on regulator proteins important for neurogenesis, including the homeobox protein Meis2 and the transcription factor Forkhead box protein G1 (FOXG1) (Shibata et al., 2008, 2011). Other miRNAs shown to regulate neuronal lineage commitment include members of the let-7 family and miR-125b (Leucht et al., 2008; Rybak et al., 2008). More recently, hippocampus-expressed miRNAs such as miR-134 and miR-34 have been shown to target the deacetylase sirtuin-1 (SIRT1) and thereby influence learning and memory processes (Gao et al., 2010; Zovoilis et al., 2011). In addition to their role in neuronal development and function, miRNAs may also play key role in neuronal dysfunction associated neurodegenerative diseases. For example, miR-34c has been implicated in the cognitive impairment associated with dementia. As discussed below, miRNAs have also been implicated in drug addiction, considered by many to be an aberrant form of learning and memory.

## Drug Addiction and MicroRNAs

Addiction can be defined as compulsive drug use despite negative consequences. During the last decade, multiple cellular and molecular studies have revealed significant convergence between the actions of drugs of abuse in the brain that drive the development of addiction and the molecular processes involved in learning and memory. Indeed, addiction is often conceptualized as a disorder of synaptic plasticity, and hence the cellular and molecular mechanisms involved in learning-associated synaptic plasticity and concomitant remodeling of neuronal circuits may provide an important heuristic framework to investigate the addiction process.

In a study by Schaefer et al. it was shown that cocaine-induced robust alterations in the expression of a wide-range of miRNAs in the striatum, a key brain site involved in addiction. Indeed, a subset of these miRNAs whose expression was impacted by cocaine were shown to regulate the expression levels of a wide-range of genes known to influence the motivational properties of cocaine, including *Bdnf*, *FosB* (*FBJ murine osteosarcoma viral oncogene homolog B*), and *Cdk5r1* (cyclin-dependent kinase 5 activator 1). In the same study, the effects on cocaine reinforcement of selectively ablating Ago2, the catalytic component of RISC involved in transducing the inhibitory actions of miRNAs on their target transcripts, was assessed. Specifically, Schaefer et al. investigated the effects knocking down Ago2 in medium spiny neurons (MSNs) of the striatum that express the dopamine D2 receptor (D2R) (Schaefer et al., 2010). Disruption of Ago2 in D2R MSNs resulted in dramatically reduced conditioned rewarding effects of cocaine in mice, reflected in attenuated cocaine-induced conditioned place preference (CPP). More importantly, the Ago2-D2R mutant mice also demonstrated reduced intravenous cocaine self-administration behavior across a wide-range of cocaine doses (Schaefer et al., 2010). Such downward shifts in the cocaine dose-response curve are interpreted as reduced motivation to consume the drug. Finally, Ago2 ablation in D2R MSNs dramatically decreased miRNA expression and activity in striatum. Hence, as cocaine self-administration behavior is considered the most direct measure of drug reinforcement in laboratory animals, these data provide compelling support for a key role for Ago2, and by extension miRNAs, in striatal MSNs in regulating the reinforcing properties of cocaine that drive the development of addiction (Schaefer et al., 2010).

Another interesting study performed by Eipper-Mains et al. (2011) links cocaine exposure to Ago2 induction (Eipper-Mains et al., 2011). Characterization of the subcellular fractions of the striatum shows that Ago2 is localized in synapses and is cocaine-responsive. Specifically, chronic cocaine exposure resulted in increased Ago2 mRNA and protein in the striatum, and concomitantly altered miRNA expression levels. This increase in Ago2 protein was associated with postsynaptic densities (PSDs) in striatum but not in medial prefrontal cortex (mPFC). Intriguingly, a large number of cocaine-responsive miRNAs identified so far (miR-8 family, miR-145, miR-451) can putatively target genes implicated in addiction, including TrkB receptor, which transduces the actions of BDNF in brain and play a crucial role in activity-dependent synaptic plasticity.

Chandrasekar and Dreyer identified another set of miRNAs (miR-181a, let-7d, and miR-124) whose expression is sensitive to cocaine (Chandrasekar and Dreyer, 2009). They found that chronic cocaine administration suppressed the expression of miR-124 and let-7d, but induced miR-181a in the mesolimbic dopaminergic system. The critical role of the mesolimbic dopaminergic system in addiction is well established (Koob and Volkow, 2010). *In situ* hybridization confirmed that the alterations in the expression of these miRNAs occurred in brain regions related to reward and memory. Further, *in vitro* overexpression of these miRNAs modulated the expression levels of proteins like BDNF and the dopamine D3 receptor, which have been heavily implicated in drug addiction (Heidbreder et al., 2005; Ghitza et al., 2010). In a subsequent study the same group also showed that *in vivo* modulation of these miRNAs in ventral striatum (nucleus accumbens) affects cocaine-induced place conditioning (Chandrasekar and Dreyer, 2011). These data show that cocaine can impact the expression of a range of different miRNAs depending on treatment and testing context, and support an important role for such miRNAs and their targeted mRNA transcripts in the development of drug addiction.

Alterations in dopamine signaling can lead to long-lasting neuronal adaptations that result in decreased or increased propensity for drug use. One of the proposed mechanism by which this can occur is dopamine transmission-induced alterations in the expression and subunit composition of AMPA receptors (Wolf, 2010). AMPA receptors are postsynaptic glutamate gated ion channels that mediate excitatory neurotransmission in the central nervous system. As such, AMPA receptors are core regulators of synaptic plasticity and activity-dependent remodeling of brain circuitries (Du et al., 2004; Haas et al., 2006). In a recent study by Saba et al. it was found that miR-181a expression in nucleus accumbens was increased by dopamine-mediated transmission and by the psychomotor stimulant drugs cocaine and amphetamines (Saba et al., 2012). Moreover, miR-181a was shown to repress GluA2-AMPA receptor subunit expression, and thereby modulate the magnitude of AMPA receptor clustering. Hence, miR-181a may be a key miRNA involved in drug-induced remodeling of the nucleus accumbens and greater striatal complex in response to drug exposure, thereby driving regulating the emergence of addiction. Recent evidence suggests that miRNAs also play a key role in the actions of other classes of addictive drugs, including nicotine, alcohol, and opiates; for recent review and detailed table describing specific miRNAs see Im and Kenny (2012).

## The miR-212/132 Cluster

**Figure 1:**
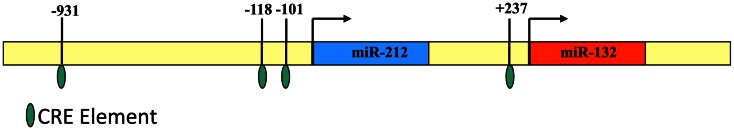
**The miR-212/132 gene cluster is located on chromosome 17 in humans, 10 in rats, and 11 in mouse**. Shown are mouse/human miR-212 and miR-132 genes, with locations of CRE elements through which CREB can stimulate miR-212 and miR-132 transcription.

The miR-132/miR-212 family of miRNAs was first identified in a genome wide search for genes responsive to the transcription factor cAMP response element binding protein (CREB) using an approach termed Serial Analysis of chromatin occupancy (SACO) (Impey et al., 2004). This family of miRNAs is highly conserved in vertebrates, is transcribed as a polycistronic primary transcript, and is highly enriched in the mature neurons of the forebrain (Marson et al., 2008; Hansen et al., 2010). Analysis of the promoter for this miRNA gene cluster reveals the presence of multiple cAMP response element (CRE) sites and experimental evidence verifies that these miRNAs are indeed CREB inducible (Vo et al., 2005); (see Figure 1) Recent studies from Remenyi et al. have shown that this gene cluster in fact produces four mature miRNAs, namely miR-132, miR-132*, miR-212, and miR-212*, where miR-132* and miR-212* are encoded by the same primary transcript, but on the opposite strand, as the miR-132 and miR-212 miRNAs, respectively (Remenyi et al., 2010). Moreover, each of these four miRNAs likely have their own unique set of target mRNA transcripts. Intriguingly, even though these four miRNAs are transcribed in equal measure (by virtue of all being encoded in the same primary transcript), their relative abundance varies dramatically within various cell types including neurons, with miR-132 being far more abundant than the other three transcripts (Remenyi et al., 2010). Hence, it is likely that as yet uncharacterized mechanisms are involved in preferentially stabilizing miR-132 levels, and/or destabilizing miR-132*, miR-212, and miR-212*.

A series of studies in the last few years have demonstrated the importance of miR-132 cluster in neuronal morphogenesis and in regulating synaptic plasticity. In particular, miR-132 has been shown to increase dendritic spine complexity in both immature cortical and hippocampal neurons in part by translational inhibition of p250GAP. As p250GAP is a Rho-Rac family GTPase activating protein, this finding highlights a role for Rho, and also transducers downstream of Rac-GTPases such as PAK, in the effects of miR-132 on activity-dependent neuronal remodeling (Wayman et al., 2008; Hansen et al., 2010; Magill et al., 2010).

## Cocaine Intake and miR-212

**Figure 2:**
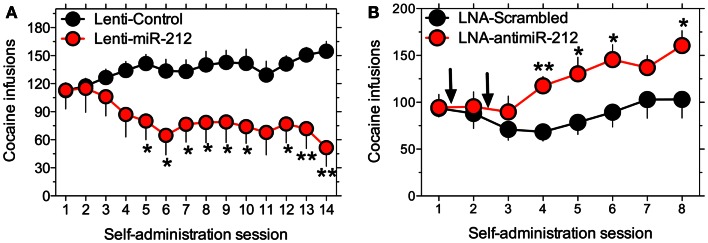
**Overexpression of miR-212 in striatum reverses the motivational properties of cocaine in rats with extended but not restricted access to cocaine**. **(A)** Striatal miR-212 overexpression reverses the long-term trajectory of cocaine-taking behavior in rats with extended access. **(B)** Disruption of miR-212 signaling in striatum, achieved by local infusion of a locked nucleic acid (LNA) modified antisense oligonucleotide against miR-212 (LNA-antimiR-212) increases cocaine intake in extended access. Reproduced with permission from (Hollander et al., 2010).

Two recent studies from our group have identified a key role for miR-212, also encoded by the miR-212/132 gene cluster, in regulating compulsive-like cocaine intake in rats (Hollander et al., 2010; Im et al., 2010). As described above, the striatum is a key brain region that regulates compulsive cocaine use. The first study showed that in rats with extended access to cocaine (6 h per day) there is a ∼1.75-fold increase in both striatal miR-212 and miR-132 levels (Hollander et al., 2010). Similar increases in expression were not detected in rats that received non-contingent cocaine infusions time-locked to rats that volitionally consumed cocaine, or in rats with restricted access to cocaine (1 h per day). Further, lentivirus-mediated overexpression of miR-212 in the dorsal striatum resulted in a remarkable decrease in cocaine intake in the extended access rats compared to vector control, but overexpression did not alter cocaine intake in rats with restricted drug access (Figure 2). The decreased cocaine intake is related to a profound decrease in the motivational properties of the drug, as reflected by a large downward shift in the cocaine dose-response curve in the same animals. Conversely, inhibition of miR-212 signaling in the striatum, achieved by infusion of an antisense oligonucleotide, dramatically increased the motivational properties of cocaine in rats with extended, but not restricted, access to the drug (Figure 2). These data suggest that intrinsic or drug-induce alterations in the expression or activity of striatal miR-212 may influence vulnerability to addiction in human cocaine users.

## miR-212 Regulates Cocaine Intake through Striatal CREB Signaling

As noted above, expression of miR-212 is regulated by CREB. In many cases, miRNAs have been shown to influence signaling cascades that increase their expression and further modify the activity those cascades through positive or negative feedback mechanisms (Tsang et al., 2007). As CREB overexpression in ventral striatum is known to diminish the motivational properties of cocaine (Carlezon et al., 1998), we tested the hypothesis that miR-212 may regulate cocaine intake in extended access rats by amplifying striatal CREB activity through positive feedback mechanisms. Consistent with a profound stimulatory effect of miR-212 on CREB in cultured cells *in vitro*, we found that levels of CREB that was phosphorylated at serine 133 (i.e., activated CREB) was significantly increased (Hollander et al., 2010). Furthermore, forskolin-stimulated expression of the CREB-responsive gene *fos* was also increased by miR-212, as was the activity of a luciferase-based CREB reporter construct (CRE-containing element from promoter of EVX-1). Dominant negative or phosphorylation-deficient mutant forms of CREB attenuated these stimulatory effects of miR-212 on CREB signaling. More importantly, we also found that miR-212 amplified CREB signaling in the striatum *in vivo*. Specifically, we found that rats with extended access to cocaine showed increased expression of the CREB-responsive gene *Nurr1*, and that expression levels were greatly increased by miR-212 overexpression in striatum (Hollander et al., 2010).

These findings identify miR-212 as a novel cocaine-responsive gene that is up regulated in the striatum in response to cocaine overconsumption that serves to promote CREB activity through positive feedback and thereby attenuate the motivational properties of the drug. Based on these findings, we next investigated the mechanisms by which miR-212 may amplify CREB signaling. We found that miR-212 increases activity-dependent production of cAMP by sensitizing adenylyl cyclase activity. This stimulatory action on adenylyl cyclase results in accumulation of phosphorylated CREB and increased activity of the core CREB co-activators CREB-regulated transcription co-activator-1 and -2 (CRTC1 and CRTC2), also known as TORC1 and TORC2. Further investigation of the mechanism by which miR-212 enhances CREB signaling revealed that the kinase Raf1 was activated by miR-212, and was found to play a key role in sensitizing adenylyl cyclase activity. Finally, computational and biochemical analysis showed that Sprouty-related EVH1 domain-containing protein 1 (SPRED1), a known negative regulator of Raf1 signaling, is a miR-212 target mRNA transcript and SPRED1 repression by miR-212 contributes to its stimulatory effects on Raf1 and CREB activity (Hollander et al., 2010).

To investigate the functional relevance of miR-212-induced amplification of CREB activity *in vivo* on the suppressive effects of this miRNA on cocaine intake, we examined the effects of overexpressing the CREB co-activator CRTC1 (TORC1) on cocaine intake in rats with restricted or extended cocaine access. It is known that CRTC overexpression increases CREB activity and that miR-212 increases CRTC1 expression (Hollander et al., 2010). Consistent with an important role for striatal CREB signaling in attenuating the motivational properties of cocaine, we found that striatal TORC1 overexpression decreased cocaine intake in extended but not restricted access rats. These findings support the hypothesis that miR-212 controls cocaine intake at least in part by amplifying the CREB-TORC signaling axis in striatum. Moreover, these findings provide compelling support for a key role for miR-212 in regulating the development of compulsive drug taking in rats, and perhaps in influencing vulnerability to cocaine addiction in human drug users.

## miR-212 also Regulates Cocaine Intake through Striatal MeCP2

**Figure 3:**
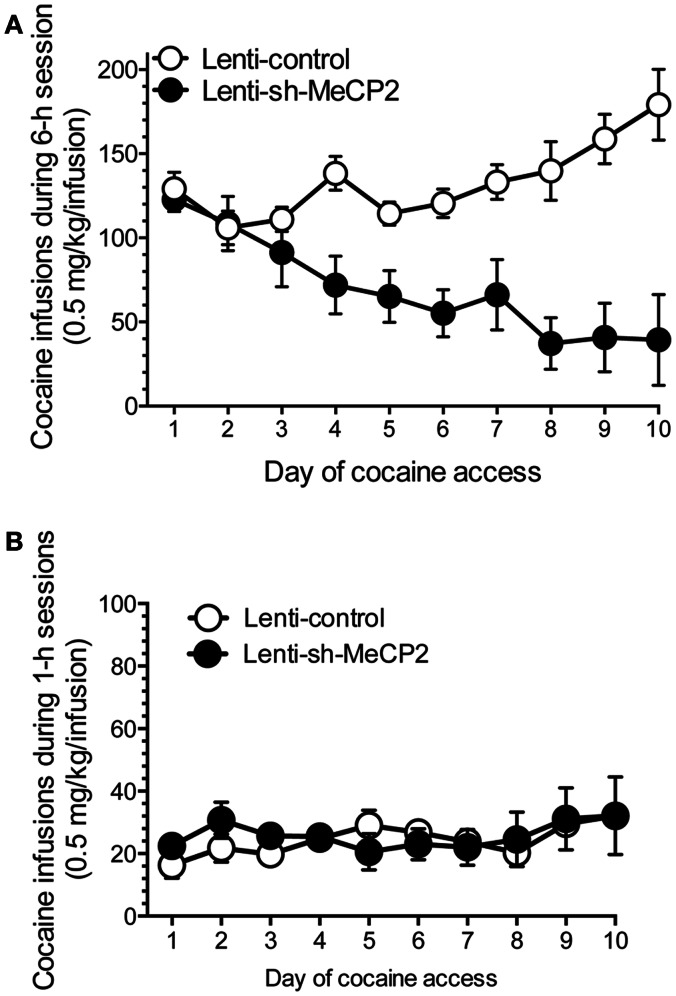
**Knockdown of MeCP2 in striatum reverses the motivational properties of cocaine in rats with extended but not restricted access to cocaine**. **(A)** Lentivirus-mediated knockdown of MeCP2 in the striatum reverses the escalating cocaine intake typically seen in rats with extended access to cocaine. **(B)** In contract, MeCP2 knockdown does not alter cocaine intake in rats with restricted daily access to the drug. Reproduced with permission from (Im et al., 2010).

Considering that miR-212 expression levels may play a key role in regulating vulnerability to cocaine addiction, we next investigated the mechanisms by which baseline and cocaine-induced increases in striatal miR-212 levels are regulated. Interestingly, sequence analysis of the miR-212/132 gene cluster reveals that it is located in a CpG enriched region, which can serve as substrate for DNA methylation and gene regulation. Methyl CpG binding protein 2 (MeCP2) is known to bind to methylated DNA and can act as a gene repressor by recruiting other chromatin remodeling proteins that combine to form the so-called repressor complex (Guy et al., 2011). Based on these observations, we hypothesized that MeCP2 may regulate baseline and cocaine-induced changes in miR-212 expression in striatum and thereby influence cocaine-taking behavior. Consistent with this hypothesis, *in vitro* studies showed that knockdown of MeCP2 increased miR-212 (and miR-132) expression in cultured cells (Im et al., 2010). Similarly, pharmacologically induced disruption of DNA methyltransferase activity, which would be expected to attenuate the inhibitory activity of MeCP2 on gene expression, also increased miR-212/132 levels. Furthermore, we found that knockdown of striatal MeCP2 expression, achieved by virus-mediated delivery of a short hairpin interfering RNA (shRNA) against MeCP2, resulted in profoundly decreased cocaine intake in rats with extended but not restricted access to cocaine (Figure 3). Knockdown of striatal MeCP2 dramatically increased the stimulatory effects of self-administered cocaine on striatal miR-212 expression in rats with extended but not restricted access to the drug. More importantly, disruption of striatal miR-212 signaling, achieved by striatal infusion of an antisense oligonucleotide, reversed the inhibitory effects of MeCP2 knockdown on cocaine intake in extended access rats (Im et al., 2010). These findings are consistent with an inhibitory effect of MeCP2 on miR-212 expression, suggesting that MeCP2 acts as a pro-addiction transcriptional repressor that, by attenuating miR-212 expression in response to cocaine, increases vulnerability to addiction.

Intriguingly, miR-132 was previously shown to repress the expression of MeCP2 through direct interaction with the transcript 3′UTR (Klein et al., 2007). Moreover, as miR-132 and miR-212 share the same seed region, this suggests that miR-212 may similarly repress MeCP2. Based on these observations, we hypothesized that in addition to the inhibitory effects of MeCP2 on miR-212 expression described above, MeCP2 levels in turn may be repressed by miR-212. In other words, miR-212 and MeCP2 may be locked in a homeostatic relationship that serves to control miR-212 expression level and thereby influence vulnerability to cocaine addiction. Consistent with this hypothesis, we found that miR-212 overexpression profoundly decreased MeCP2 expression in cultured cells and in the striatum *in vivo* (Im et al., 2010). Hence, homeostatic interactions between miR-212 and MeCP2 may determine vulnerability to cocaine addiction.

## Role for BDNF in Regulating the Actions of Striatal miR-212 on Cocaine Intake

It has been reported that the levels of MeCP2 are closely related to those of, BDNF (Chang et al., 2006). However, the complex mechanisms by which MeCP2 regulates BDNF levels remain unclear. BDNF in the striatum is known to increase the motivational properties of cocaine (Horger et al., 1999; Schoenbaum et al., 2007). We therefore hypothesized that miR-212-MeCP2 interactions may regulate cocaine intake by influencing levels of BDNF in striatum. We observed that virus-mediated MeCP2 knockdown or miR-212 expression (which decreases MeCP2 levels) in striatum reduced BDNF expression. Moreover, virus-mediated increases in BDNF expression in striatum increased cocaine intake in rats with extended but not restricted access. Conversely, disruption of striatal BDNF signaling using a neutralizing antibody reduced cocaine intake in extended but restricted access rats (Im et al., 2010). These findings suggest that miR-212-MeCP2 interactions may determine expression levels of BDNF in striatum, which in turn regulates the motivational properties of cocaine.

## Non-Neuronal Roles of miR-212

Besides playing a critical role in drug-induced neuroplasticity relevant to addiction, there is emerging evidence that miR-212 is also involved in a host of other biological and pathophysiological processes. Indeed, miR-212 expression is deregulated in various cancers and its expression has been correlated to disease progression. In pancreatic carcinomas it has been shown that miR-212 targets the tumor suppressor retinoblastoma (Rb1) (Park et al., 2011), whereas the methyl binding protein (MeCP2) seems to be the main target in some of the gastric tumors (Wada et al., 2010). Incoronato et al. showed that miR-212 targets phosphoprotein enriched in diabetes (PED), a wide spectrum anti-apoptotic protein, and thereby plays an important role in tumor suppression (Incoronato et al., 2010). In a follow up study the same group also demonstrated that the expression on miR-212 in lung carcinomas is regulated by histone modifications (Incoronato et al., 2011). This suggests that epigenetic mechanisms may play an important role in regulating miRNA expression in different cancers and other biological processes.

Besides cancer, miR-212 has also been implicated in regulating organogenesis by playing a key role in modulating epithelial stromal interactions (Ucar et al., 2010). In addition, miR-212 expression can be regulated by various hormones, adding another layer of complexity to their regulation and role in development and disease (Godoy et al., 2011). Studies from Turrini et al. reveal that miR-212 may mediate drug resistance by targeting the ABC efflux transporter (Turrini et al., 2012). Two recent reports implicate the importance of the miR-132/212 family in cardiovascular development and disorders. In the first study, smRNAs deep sequencing analysis in vascular smooth muscle cells show that the miR-132/miR-212 cluster is induced by the hormone angiotensin II and by targeting PTEN, increases the expression of the gene MCP1 (Monocyte chemotactic protein 1), a key regulator of cardiovascular disorders (Jin et al., 2012). In the other study, Ucar et al. demonstrate that these miRNAs activate calcineurin signaling in cardiomyocytes by targeting the transcription factor Foxo3, and thus play an important role in cardiac hypertrophy (Ucar et al., 2012).

## Conclusion

Taken together, these studies highlight the fact that miR-212 plays a critical role in fine-tuning transcriptional and neuroplastic responses to drugs of abuse. Specifically, we found that miR-212 can control cocaine intake through two complementary mechanisms: amplifying CREB signaling and reducing MeCP2/BDNF transmission in striatum.

Since miR-212 and miR-132 share the same seed region it is widely believed that they target the same mRNAs. However, only a few putative miR-212/132 targets have been verified experimentally, which include MeCP2, Rb1and HB-EGF. There is increasing evidence available now showing that the 5′ and 3′ regions of the miRNAs can form the basis of differential target recognition despite having the identical seed region (Brennecke et al., 2005; Jalvy-Delvaille et al., 2012). There is also a possibility that differential expression or availability of these members can also regulate gene expression in a differential manner. Future studies in this respect could further help in improving our understanding of how miR-212 and miR-132 regulate different neuronal and non-neuronal functions or if these two miRNAs are functionally redundant? More generally, these findings highlight the novel role for miRNAs in addiction, and suggest that other ncRNAs may also play important roles in the disorder.

## Conflict of Interest Statement

The authors declare that the research was conducted in the absence of any commercial or financial relationships that could be construed as a potential conflict of interest.
